# HLA-DQ B1*0201 and A1*0102 Alleles Are Not Responsible for Antituberculosis Drug-Induced Hepatotoxicity Risk in Spanish Population

**DOI:** 10.3389/fmed.2016.00034

**Published:** 2016-08-22

**Authors:** Virginia Leiro-Fernández, Diana Valverde, Rafael Vázquez-Gallardo, Lucía Constenla-Caramés, Víctor del Campo-Pérez, Alberto Fernández-Villar

**Affiliations:** ^1^NeumoVigol+i Research Group, Pulmonary Department, EOXI Vigo, Biomedical Research Institute Vigo (IBIV), Alvaro Cunqueiro Hospital, Vigo, Spain; ^2^Biochemical, Genetics and Immunology Department, Biology Faculty, Vigo University, Vigo, Spain; ^3^Clinic Analysis Department, EOXI Vigo, Alvaro Cunqueiro Hospital, Vigo, Spain; ^4^Preventive Medicine Department, EOXI Vigo, Alvaro Cunqueiro Hospital, Vigo, Spain

**Keywords:** DQB1*0201, DQA1*0102, hepatitis, pharmacogenetics susceptibility, first-line antituberculosis drugs

## Abstract

**Aims:**

To evaluate the role of human leukocyte antigen (HLA) class II DQB1*0201 and DQA1*0102 in the risk of antituberculosis drug (ATD)-induced hepatotoxicity (ATDH) in a cohort of tuberculosis patients of Caucasian origin from Spain.

**Methods:**

Matched case-control study including active tuberculosis (TB) patients from Spain (Caucasian) treated with first-line ATD (Isoniazid, Rifampin, and Pyrazinamide). Presence or absence of HLA class II DQB1*0201 and DQA1*0102 alleles were compared between cases and controls.

**Results:**

We included 110 TB patients, 55 ATDH cases, and 55 sex-matched controls. The analysis of the presence of HLA-DQB1*0201 and HLA-DQA*0102 did not show significative differences between both groups [presence of HLA-DQB1*0201 53.6% of the cases vs. 45.4% of the controls, OR: 1.63 95% CI (0.62–4.52) *p* = 0.38; presence of HLA-DQA*0102 7.5% of cases vs. 20% of controls, OR: 0.36 95% CI (0.08–1.23) *p* = 0.12]. After multivariate logistic regression analysis including in the model, other potential risk factors of hepatotoxicity HLA class II DQB1*0201 and DQA1*0102 alleles were not found significantly associated with the risk of development ATDH. We could not demonstrate an association between HLA-DQA1*0102 and HLA-DQB1*0201 with the risk of ATDH in this Caucasian population of Spanish origin.

## Introduction

Tuberculosis (TB) continues to be a global health problem despite the availability of effective therapies. Hepatotoxicity is one of the most important side effects of first-line antituberculosis drugs (ATD) that could lead to increase in morbidity disease because of treatment interruptions and negative impact in treatment compliance including increasing costs (1, 2). The first-line ATD-induced hepatotoxicity (ATDH) is isoniazid (INH), rifampicin (RIF), and pyrazinamide (PZA). Many risk factors for ATDH have been reported, and its incidence depends on the number of risk factors, varying from 5.8 to 24.2% (1–5). Genetic susceptibility to ATDH has been the subject of study during last years focusing in genes encoding phase I and II metabolic enzymes, and the risk has been significantly associated with certain genetic polymorphisms (3, 6–11). Human leukocyte antigen (HLA) class I and II drug-induced hepatotoxicity associations have been identified with other drugs and also in healthy individuals with alanine transaminase (ALT) elevations (7). Each HLA associations points to a role for an immune mechanism. Even that the majority of recently published studies on ATDH have been concerned with polymorphisms affecting drug-metabolizing enzymes, there are evidence that some HLA class II alleles are related to the risk of ATDH in the Indian and Chinese population (12, 13). The purpose of this study is to evaluate the risk of ATDH related to HLA class II DQB1*0201 and DQA1*0102 in a cohort of TB patients of Caucasian origin from Spain.

## Materials and Methods

### Study Design

This individually matched case-control study was nested in a cohort of 1,200 patients from Caucasian origin with active TB that had been treated between January 1998 and December 2008 at the TB Units in Complexo Hospitalario Universitario de Vigo, Pontevedra, and A Coruña (Galicia, Spain). Some characteristics of this cohort were reported in previous publications (5, 8, 9). We included all cases of ATDH that fulfilled the inclusion criteria. The control group consisted of sex- and drug-matched patients that showed no evidence of ATDH, selected consecutively from the same cohort. The study participants should follow the inclusion criteria: (a) age between 15 and 75 years old, (b) microbiological demonstration of active TB, (c) treatment with regimens that included INH, RIF, and PZA at the usual drug dosages (INH 5 mg/kg/day – maximum 300 mg/day, RIF 10 mg/kg/day – maximum 600 mg/day, and PZA 25–30 mg/kg/day – maximum 2,500 mg/day), and (d) adequate compliance. Exclusion criteria were: (a) increased baseline serum transaminases [aspartate aminotransferase (AST) and/or alanine aminotransferase (ALT)], normal values ≤40 IU/L, (b) positive serological testing for the human immunodeficiency virus, hepatitis B virus, or hepatitis C virus, (c) regular alcohol intake or concomitant use of hepatotoxic drugs, (d) history of chronic liver disease, (e) pregnancy, or (f) no or poor adherence to treatment.

### Diagnosis of Antituberculosis Drug-Induced Hepatotoxicity

Antituberculosis drug-induced hepatotoxicity was defined as an increase in serum transaminase (either aspartate aminotransferase or alanine aminotransferase) to values higher than three times the upper limit of normal (ULN) (i.e., >120 IU/L) at any time during the treatment period (1). The degree of severity of hepatotoxicity was gaged by the peak level of serum transaminases, and classified according to the Toxicity Classification Standards (10).

### Standard Follow-up of Patients on Antituberculosis Drug Therapy

In this cohort, patients on antituberculosis drug therapy received a routine follow-up of clinical assessments every 2 weeks during the first month, and thereafter, monthly until completion of therapy. Routine liver function tests [blood cell counts and biochemical assays for serum AST, ALT, gamma glutamyl transpeptidase (GGT), and bilirubin (BIL)] were performed 15–30 days after the beginning of treatment, at the end of 2 and 4 months, and at 2-month intervals thereafter, when treatments lasted for more than 6 months. Clinical and laboratory check-ups were performed more frequently when hepatitis symptoms or abnormal serum transaminase levels were observed. All patients were warned verbally and in writing of possible hepatotoxicity symptoms and were provided with an emergency contact telephone number. Adherence to treatment was verified by detection of INH metabolites in urine specimens and by tablet counts at every visit.

### Genotyping Protocol

Blood was collected by venipuncture, and genomic DNA was isolated with Flexi Gene DNA Kit (QIAGEN) following the instructions of manufacturer (Supplementary Material). The HLA class II region (second exon) was amplified by polymerase chain reaction (PCR). Specifically DQB1*0201 exon 2 genotype was detected by PCR as previously described with minor modifications (14, 15).

### Statistical Analyses

Qualitative variables were expressed as percentages and absolute frequencies, and quantitative variables as the median and interquartile range (IQR) or median and SD. Qualitative variables were compared using the McNemar test. Quantitative variables were analyzed using the Wilcoxon test. A logistic regression model was developed in order to evaluate the risk of ATD-induced hepatitis in the presence or absence of HLA DQB1*0201 and DQA1*0201 alleles. Variables were adjusted for potential confounders, including age (in years), sex, nutritional status evaluated by the body mass index [BMI = weight/(height in meters)^2^], and baseline transaminase values. The patient’s sample size was determined from allele frequencies observed for healthy individuals with a genetic model analyzing the frequency for the disease gene with a RR value = 2 (α = 0.05). The statistical power for variant alleles calculated for the sample size using healthy control population as a reference is 80% for unilateral association.^1^ A *p* value less than 0.05 was considered statistically significant.

### Ethical Issues

This investigation followed the tenets of the Helsinki declaration. It was approved by the Ethical Committee of Galicia (CEIC). Written informed consent was obtained from all participants. All participants were adults aged more than 18 years.

## Results

We included 110 TB patients, 55 with ATDH, and 55 controls with no evidence of this complication. The baseline characteristics are showed in Table 1. Hepatotoxicity was mild in 34% [median peak (IQR) of ALT, AST, GGT, and BIL of 154 IU/L (133–181), 94 IU/L (72–117), 14 IU/L (30–59), and 0.4 mg/dL (0.3–0.65), respectively], moderate in 42.6% [median peak (IQR) of ALT, AST, GGT and BIL of 260 IU/L (220–337), 130 IU/L (108–198), 87 IU/L (46–120), and 0.45 mg/dL (0.3–0.53), respectively] and severe in 23% [median peak (IQR) of ALT, AST, GGT, and BIL of 953.5 IU/L (699–1126), 647 IU/L (239–826), 73 IU/L (61.2–139), and 0.45 mg/dL (0.3–1.01), respectively]. The median length of time from the beginning of treatment until the development of hepatotoxicity was 45 days (IQR, 22–69 days).

**Table 1:** **Baseline characteristics of cases and controls**.

	Cases	Controls	*p*
Age (years)	41 (15.8)	36.3 (15.5)	<0.001
BMI (kg/m^2^)	23.5 (3.1)	22.8 (3.4)	0.12
Serum ALT (IU/L)	22 (7.7)	20.1 (9)	0.38
Serum AST (IU/L)	21.6 (5.8)	21 (6.6)	0.38

*Variables are expressed as mean and SD (in parentheses). BMI, body mass index; ALT, alanine-transaminase; AST, aspartate transaminase; Normal intervals of serum ALT: 4–40 IU/L; AST: 4–40 IU/L. IU, International units*.

*Cases: patients with ATDH*.

*Controls: patients with no evidence of ATDH*.

### HLA Analysis

HLA DQA*0102 analysis was completed in 53 ATDH patients and in 53 controls. The frequency of the presence of HLA DQA*0102 was 7.5% in ATDH patients and 20% in non-ATDH patients with no significant differences between cases and controls (OR: 0.36 95% CI 0.08–1.23, *p* = 0.12) (Table 2). The analysis of the presence of HLA DQB1*0201 allele, completed in 110 patients, did not show differences between both groups of ATDH cases and non-ATDH controls [53.6 vs. 45.4%, respectively, OR: 1.63 95% CI (0.62–4.52) *p* = 0.38] (Table 2). When we performed the analysis by hepatotoxicity degree, we did not show differences in HLA DQA*0102 and HLA DQB1*0201 presence in the different subgroups (mild, moderate, and severe hepatotoxicity). After multivariate logistic regression analysis including in the model, other potential risk factors of hepatotoxicity as advancing age, BMI, and pre-treatment transaminase values alterations in HLA class II DQB1*0201 and DQA1*0102 alleles were not found significantly associated with the risk of development ATDH.

**Table 2:** HLA II DQB1*0201 and DQA1*0102 analysis in patients with TB and with or without hepatotoxicity.

	HLA DQB1*0201 (presence)	HLA DQA1*0102 (presence)
Cases	30/55 (53.6%)	4/53 (7.5%)
Controls	25/55 (45.4%)	11/53 (20%)
*p*	0.38	0.12
OR, CI 95%	1.63, 0.62–4.52	0.36, 0.08–1.23

*OR, odds ratio; CI, confidence interval*.

*Cases: patients with ATDH*.

*Controls: patients with no evidence of ATDH*.

## Discussion

This study performed in Caucasian origin TB patients showed no significant association between HLA DQA1*0102 absence and the risk of hepatotoxicity when the first-line standard therapy is used (INH, RIF, and PZA). However, there is a non-significant predominance of its absence in cases (92.5 vs. 80%). We also did not find an increased ATDH risk in HLA DQB1*0201 carriers. Most of the genetic risk in ATDH is focused in genes encoding drug-metabolizing enzymes that could explain interindividual variation in response and toxicity with ATD (6–11). However, other pathways of adverse drug reactions like hypersensitivity reactions should be deeply studied in ATDH. Different hypothesis may explain immune-mediated reactions, and the major histocompatibility complex (MHC) class I or II molecules are always involved due to its crucial role in immune recognition (11). Interestingly, HLA variants have been linked to autoimmune hepatitis (7, 11, 12). Association of HLA variants has been described with different drugs as amoxicilin-clavulanate (16, 17), flucoxacilin (11), ximelagatran (11), lapatinib (18), lumiracoxib (19, 20), and neviparine (21). Sharma et al. showed a fourfold increased risk of ATDH in patients without HLADQA1*0102 haplotype suggesting to play a role in pathogenic development of ATDH (12).

Our investigation did not show association in an ethnically different group of TB patients, with a Caucasian origin from Spain. In a case-control study from China, exploring *HLADQB1* alleles found that only DQB1*05/*05 positive genotype patients were in risk of ATDH after being adjusted by other protective drugs and weight. Sharma et al. also found, relevantly, the presence of HLADQB1*0201 in ATDH risk. In our study, these finding were not replicated too. An explanation could be the different ethnical origin of Sharma et al. study population. Differences in HLA allele associations have been recently demonstrated between ethnical groups (7). There are few data to support an association between HLA alleles and ATDH risk; however, the results from the studies are heterogeneous probably due to small sample sizes and different ethnical origin (7).

Singer et al. have been described for the selective cyclooxygenase-2 inhibitor lumiracoxib an increased risk of liver disease secondary to lumiracoxib treatment linked to the presence of HLAQA1*0102 allele (OR = 5.0, 95% CI 3.6–7.0) (19). Probably, differences could be explained by the different hepatotoxicity mechanism of non-steroidal drugs, and the interactions between several factors inherent to subjacent disease and the patients.

One of the most important issues is to analyze the convenience of determining HLADQA1*0102 to predict possible hepatotoxic reaction to ATD that could lead to closer clinical and analytic monitoring of TB patients. However, after our results, we cannot make specific recommendations. The purpose of our study was not to analyze its relevance but rather a possible association between HLADQA1*0102 and the risk of ATDH in absence of other previously known ATDH risk factors. For that reason, the criteria of hepatotoxicity definition is low (three times de ULN AST/ALT) and not clinically relevant in most of the cases (1). Variations in the definitions of hepatotoxicity may have given rise to different results among studies. Another potential limitation of the study was that the sample size was small. It was due to the very strict inclusion criteria that exclude TB patients with any previously known ATDH risk factor. We are aware that the failure to find differences in could be due to small sample so that the null hypothesis could be rejected when the alternative hypothesis is true (type II error). The analysis should, therefore, be replicated in a larger study with effective sample size, i.e., containing proband number that achieves adequate statistical power. Finally, we must consider the possibility of frequent bias of genetic case control. This was a nested case-control study design. However, it utilizes the exposure and the confounder data originally collected prior to the onset of the disease, thus reducing potential recall bias and temporal ambiguity (minor concerns in genetic case-control studies). Furthermore, nested studies include cases and controls drawn from the same cohort, thus decreasing the likelihood of selection bias.

## Conclusion

We can conclude that even the absence of HLADQA1*0102 could be a risk factor associated with ATDH risk, we cannot demonstrate this association in a cohort of TB patients from Caucasian origin Spanish. We also could not demonstrate an association between HLADQB1*0201 and ATDH. More genetics investigations should be performed in conjunction with the rest of the genetic and clinical ATDH risk factors to determine its routine practical application in TB patients subsidiaries of treatment with first-line ATD.

## Author Contributions

VL-F had full access to all of the data in the study and takes responsibility for the integrity of the data and the accuracy of the data analysis. Study concept and design: VL-F, DV, AF-V, and VC-P. Acquisition of data: VL-F, RV-G, and AF-V Analysis and interpretation of data: LC-C, DV, VL-F, and VC-P. Drafting of the manuscript: VL-F. Critical revision of the manuscript for important intellectual content: DV, VC-P, and AF-V. Statistical analysis: VL-F and VC-P. All authors declare that they have participated actively in the study, all the authors have read and approved the submitted manuscript, the study complies with current ethical considerations, the manuscript reports unpublished work that is not currently under consideration elsewhere and will not be submitted to another journal until a final decision has been made, and there are no conflicts of interest, unless those identified specifically by the authors in the letter.

## Conflict of Interest Statement

The authors declare that the research was conducted in the absence of any commercial or financial relationships that could be construed as a potential conflict of interest.

## Footnotes

^1^Available from: http://www.allelefrequencies.net/. Consulted May 2016.

## Abbreviations

ALT, alanine transaminase; AST, aspartate transaminase; ATD, antituberculosis drugs; ATDH antituberculosis drugs-induced hepatotoxixty; BIL, bilirubin; BMI, body mass index; CEIC, Ethical Committee of Galicia; GGT, gamma glutamyl transpeptidase; HLA, human leukocyte antigen; INH, isoniazid; IQR, interquartile range; PCR, polymerase chain reaction; PZA, pyrazinamide; RIF, rifampicin; SBT, sequence based typing; SD; TB, tuberculosis; IU, international units; ULN, upper limit of normal.
