# Supplementary material for: HLA-DQ B1*0201 and A1*0102 Alleles Are Not Responsible for Antituberculosis Drug-Induced Hepatotoxicity Risk in Spanish Population
Source: Front Med (Lausanne). 2016 Aug 22;3:34. doi: 10.3389/fmed.2016.00034 (PMC4992991; doi:10.3389/fmed.2016.00034)
Supplement: Supplementary file 1 [file datasheet_1.docx]

**Supplementary file**

**HLA genotyping method**

PCR was performed using the primers 5’-CGCGTGCGTCTTGTGAGCAGAAG-3’ and 5’-GGCGGCAGGCAGCCCCAGCA-3’ in a final volume of 25µl at the following settings: 5 min denaturation at 95ºC, followed by 30 cycles of 30 sec at 94ºC, 20 sec at 72ºC and a final 10 min extension at 72ºC. The presence or absence of HLADQB1*0201 was visualized in a 0.7% agarose gel containing 10mg/ml ethidium bromide, visualized by UV light and compared with the molecular weigth marker (Ecolader IV, 1000 bp; Bioline Ltd, London, UK). The exon 2 of the HLADQA1 gene was amplified by the polymerase chain reaction (PCR) in a MJ MiniTM Gradient Thermal Cycler (Bio-Rad). Primers used were previously described [16]. Control PCR amplification was visualized in 2% agarose gels in TAE buffer. The PCR products obtained were purified with illustraTM GFXTM MicroSpin columns (GE) following the supplier´s protocol and directly sequenced using the Big Dye Terminator (Applied Biosystems). The sequencing reactions were precipitated, dried and analyzed on an AB 3130 genetic analyzer (Applied Biosystems). We use the Sequence Based Typing (SBT) as a method for defining the HLA type, most SBT typing strategies currently employed use the exon 2 alone for HLA class II analysis (http://www.ncbi.nlm.nih.gov/gv/mhc/sbt.cgi?cmd=main).
